# Supplementary material for: Emergency mortality of non-trauma patients was predicted by qSOFA score
Source: PLoS One. 2021 Feb 24;16(2):e0247577. doi: 10.1371/journal.pone.0247577 (PMC7904145; doi:10.1371/journal.pone.0247577)
Supplement: Attachment — Submitted filename: Response to Reviewers.docx [file pone.0247577.s002.docx]

Dear Editor,

Thank you for reviewing the manuscript, we read your comments and suggestions carefully.

I'm sorry that the wording of the title of our manuscript may be inaccurate, resulting in your misunderstanding.

In fact, Paper1 (Emergency mortality of medical patients was predicted by qSOFA score) and Paper2 (Predictive value of qSOFA score for death in emergency department resuscitation room among adult trauma patients) were two separate sample studies, conducted by two different researchers (YuFang Li, WenJuan Huang) respectively. The study samples of Paper1 were non-traumatic patients (n=11621) treated by physicians in the emergency room, while the study samples of Paper2 were trauma patients (n=1739) treated by surgeons in the emergency room. Therefore, the research sample of Paper2 is not a subgroup of the research sample of Paper 1, but two different research samples.

In addition, there was considerable discrepancy between their research methods. For example, only age and gender were included in the multivariate regression model for non-traumatic patients to calculate the adjusted OR in Paper 1, but RIS scores reflecting the severity of injuries were added as adjusted variables in Paper2. Therefore, the adjusted OR meanings of these two studies are different.

To sum up, the study population of Paper1 does not include the study population of Paper2, and the research methods are also not exactly the same, so the two papers cannot be combined.

In order to avoid misunderstanding, we decided to revise the title of Paper1 as *Emergency mortality of internal medicine patients was predicted by qSOFA score.*

Thank you again for all your hard work and generous help.

Kind regards,

Du Chen
