# Supplementary material for: Emergency mortality of non-trauma patients was predicted by qSOFA score
Source: PLoS One. 2021 Feb 24;16(2):e0247577. doi: 10.1371/journal.pone.0247577 (PMC7904145; doi:10.1371/journal.pone.0247577)
Supplement: Attachment — Submitted filename: Response to Reviewers.docx [file pone.0247577.s003.docx]

**Dear Editors:**

Thank you for your good suggestions. The question of written consent from the study participants has been revised in the methods section of the manuscript.

The following is my reply to the reviewer.

**To Reviewer #1**

Dear reviewer，thank you very much for your review and suggestions.

Because the qSOFA score contains only three simple binary variables which are easy to get, we wanted to verify the predictive value of qSOFA score for the prognosis of non-trauma patients, including sepsis. The aim is to expand the scope of the qSOFA score and provide a simple assessment tool for non-trauma patients in the emergency department.

Your suggestion to compare the qSOFA score with the MEWS score has been adopted and revised in the manuscript. Our study shows that AUROC of qSOFA is lower than MEWS score, and the difference is statistically significant. But qSOFA scores have less content and are easier to calculate.The MEWS was miscalculated frequently, the probability as high as 18.2% (The 17th reference in the manuscript). Compared to MEWS, the AUC of qSOFA score is slightly lower, but still has good predictive value and can be used as a supplement to the existing triage system.

Finally, thank you for your suggestion to modify the title. We have modified the title as “Emergency mortality of non-trauma patients was predicted by the qSOFA score”.

**To Reviewer #2**

Dear reviewer，thank you very much for your review and suggestions.

--**Abstract, Discussion:** It is true, as you said, that although the qSOFA score is associated with ED mortality in non-trauma patients and shows a good prognostic performance, this study is just a retrospective study, a prospective will be required.

--**Abstract, Materials and Methods:** Your suggestion is very constructive, the concept of internal medical patients is really vague, we have modified the title as ”Emergency mortality of non-trauma patients was predicted by the qSOFA score”.Our aim is to expand the practicability of qSOFA score , especially in undifferentiated non-trauma patients, so we didn’t conduct a subgroup analysis based on medical pathologies.

--**Abstract, Materials and Methods:** The purpose of our study was to verify the predictive value for the prognosis of non-traumatic patients，expand the scope of qSOFA score and provide a simple assessment tool for all non-trauma patients in the emergency department. And studies have confirmed that the predictive validity of death in patients with and without suspected infection were similarly high (The 12th reference in the manuscript). So we did not deliberately distinguish between the infected and non-infected patients.

-- **Materials and Methods:** All the non-trauma patients included in this study were treated by physicians, while neurological diseases were treated by neurologist, so they were not included in the study.

-- **Materials and Methods:** This is a retrospective study that extracted information from the hospital emergency electronic record system. The time of onset, complications, ICU admission, and other information were not recorded. To sum up, this study does have some limitations in design.

-- **Materials and Methods:** The Cox regression you proposed instead of logistic regression was indeed very good. However, since our system only has the time of register, we cannot accurately calculate the time from onset to death. After careful consideration, logistic regression analysis was still used.

-- **Materials and Methods,** **Results:** The only outcome of our study was ED mortality, so we did not consider post-admission deaths and ICU admissions of non-traumatic patients.

-- **Results, Materials and Methods, Discussion:** In the discussion section, we have compared the results with those of some published studies, especially those that, like our studies, target people who are not sepsis(References to articles 6-8 and 12-13 in the manuscript). The predictive performance of qSOFA score and MEWS score was also compared in the results and discussion section.

Best Regards.

Yours Sincerely,

Du Chen
