# Supplementary material for: Emergency mortality of non-trauma patients was predicted by qSOFA score
Source: PLoS One. 2021 Feb 24;16(2):e0247577. doi: 10.1371/journal.pone.0247577 (PMC7904145; doi:10.1371/journal.pone.0247577)
Supplement: Attachment — Submitted filename: Response to Reviewers.docx [file pone.0247577.s004.docx]

**Dear Marleen Smits, PhD:**

Thank you for your work. The following is my reply to the reviewer.

**To Reviewer #1**

Dear reviewer，thank you very much for your review and suggestions.

We have calculated best cutoff values, sensitivity and specificity for qSOFA score and MEWS score based on the Yoden index (listed in the results section of the manuscript).

Indeed, due to the widespread use of computers, the complexity and error-prone nature of MEWS score are negligible problems, so we clearly suggest that MEWS was a better choice as a triage tool.

According to the results of the study,the qSOFA score can be used as a tool to predict ED mortality in non-trauma patients.

**To Reviewer #2**

Dear reviewer，thank you very much for your review and suggestions.

The English form has been deeply modified.

Best Regards.

Yours Sincerely,

Du Chen
